# Prostate Cancer Survivors’ and Caregivers’ Experiences Using Behavior Change Techniques during a Web-Based Self-Management and Physical Activity Program: A Qualitative Study

**DOI:** 10.3390/jcm9103244

**Published:** 2020-10-11

**Authors:** Laura Hallward, Keryn Chemtob, Sylvie D. Lambert, Lindsay R. Duncan

**Affiliations:** 1 Department of Kinesiology and Physical Education, McGill University, Montreal, QC H2W 1S4, Canada; laura.hallward@mail.mcgill.ca (L.H.); keryn.chemtob@mail.mcgill.ca (K.C.); 2 Ingram School of Nursing, McGill University, Montreal, QC H3A 2M7, Canada; sylvie.lambert@mcgill.ca; 3 St. Mary’s Research Centre, Montreal, QC H3T 1M5, Canada

**Keywords:** prostate cancer, cancer survivorship, cancer rehabilitation, self-management, behavior change, caregivers, dyadic intervention

## Abstract

Both men with prostate cancer and their caregivers report experiencing a number of challenges and health consequences, and require programs to help support the cancer patient–caregiver dyad. A tailored, web-based, psychosocial and physical activity self-management program (TEMPO), which implements behavior change techniques to help facilitate behavior change for the dyads was created and its acceptability was tested in a qualitative study. The purpose of this secondary analysis was to explore the dyads’ experiences using behavior change techniques to change behavior and address current needs and challenges while enrolled in TEMPO. Multiple semi-structured interviews were conducted with 19 prostate cancer-caregiver dyads over the course of the program, resulting in 46 transcripts that were analyzed using an inductive thematic analysis. Results revealed four main themes: (1) learning new behavior change techniques, (2) engaging with behavior change techniques learned in the past, (3) resisting full engagement with behavior change techniques, and (4) experiencing positive outcomes from using behavior change techniques. The dyads’ discussions of encountering behavior change techniques provided unique insight into the process of learning and implementing behavior change techniques through a web-based self-management program, and the positive outcomes that resulted from behavior changes.

## 1. Introduction

Men in North American are more likely to develop prostate cancer than any other cancer, with one in nine men expected to be diagnosed with prostate cancer in his lifetime [1,2]. Due to increased screening, early detection, and improvements in treatment options, the rate of death due to prostate cancer has been steadily declining with an estimated 10-year survival rate of 98% [1,2]. Given the high survival rates, more cancer survivors (A cancer survivor is anyone “living with, through, and beyond a cancer diagnosis” [3]) are living with the associated consequences from cancer and cancer treatment [4]. Men with prostate cancer report side effects such as urinary incontinence, sexual dysfunction, and an increased risk of cardiovascular complications, metabolic syndrome, and overall morbidity and/or mortality [5,6]. Prostate cancer survivors also experience psychological problems such as depression, anxiety, fatigue, and a compromised quality of life [7,8,9]. Due to the associated health consequences of a cancer diagnosis and cancer treatment faced by prostate cancer survivors, family and friends are taking on the role of caregiver [10].

Caregivers are defined as individuals providing unpaid care for someone with an illness, disability, or age-related issues. Over a quarter of Canadians and one fifth of Americans are considered caregivers [10], and the numbers are only expected to increase [11]. The second most common health condition requiring care is cancer (17% of the caregiver population) and most often care is provided by spouses [10,12]. Caregivers are often responsible for tasks such as providing transportation, communicating with health care providers, and performing personal tasks for the survivor such as monitoring symptoms, managing medication, and providing emotional support [13]. Caregiving has the benefit of reducing costs and demands on the health care system, and allows more cancer survivors to be cared for at home [10]. However, due to the added responsibility of caring for someone with cancer, caregivers can face numerous physical, psychological, and financial problems [10]. Caregivers often report difficulty with sleep, fatigue, pain, and loss of strength [14]. There is evidence indicating that a cancer diagnosis can cause more burden and distress for spousal caregivers than the cancer survivors themselves [14,15]. Both cancer survivors and their caregivers experience numerous physical and psychological consequences and would benefit from supportive strategies to self-manage their health. 

In light of the challenges faced by cancer survivors and caregivers, a number of interventions and programs have been developed to address the cancer survivor–caregiver dyads’ needs and improve their quality of life. The majority of interventions developed for cancer survivors and caregivers are psychoeducational whereby they provide information about symptom management and physical care for the cancer survivor [16,17,18]. Interventions may also focus on emotional and psychological needs, such as managing marital concerns, and/or include coping, communication, and problem-solving skills [17]. Dyadic interventions have been shown to improve coping abilities, self-efficacy, communication and relationship functioning, distress, and quality of life for the cancer survivors and caregivers [17,18]. Despite the positive benefits of psychoeducation for cancer survivors and caregivers, interventions often fail to address the physical health demands of the dyad.

With the recognized physical and psychological benefits of physical activity, it can provide a beneficial strategy for cancer survivors and caregivers to improve their physical health, and additionally incur psychological benefits. Among prostate cancer survivors, evidence from multiple systematic reviews examining the effects of physical activity has shown improved physical functioning, muscular and aerobic fitness, and overall quality of life, and decreased urinary incontinence and fatigue [19,20,21,22]. Among cancer caregivers, few interventions have been developed to target their physical health despite caregivers expressing the desire for such programs [23]. In a systematic review of physical activity programs for caregivers [24], only one pre-experimental study focused on cancer caregivers, and showed decreases in distress and increased aspects of quality of life [25]. Interventions that target both cancer survivors and caregivers that include not only psychoeducation, but also a physical activity component, could target the overall health of both members of the dyad. 

Given the need, our research team has developed TEMPO, a dyadic, Tailored, wEb-based, physical activity and psychosocial self-Management PrOgram designed for prostate cancer survivors and their caregivers. The objectives of TEMPO are to (a) increase the dyad’s confidence in using strategies to address key psychological issues faced by the dyad, such as anxiety, stress, and worry, and (b) assist the dyads in developing self-regulatory skills to increase physical activity levels. The web-based intervention is comprised of five modules with associated worksheets, and factsheets found in a supplemental health library. The five modules are released approximately every two weeks and provide information, worksheets, and behavior change strategies for the dyads to help them cope with psychological issues and promote physical activity. Module 1 is a dyadic needs assessment; Module 2 focuses on goal setting and action planning; Module 3 involves coping planning; Module 4 emphasizes sources of support and motivational tools; and Module 5 focuses on past successes and the next steps after completing the intervention. Another resource included in TEMPO is the health library, which provides helpful information on numerous areas relevant to the challenges faced by the dyads, such as making treatment decisions, dealing with stress and worry, or wanting to feel more fit and healthy. Participants in this study were also provided with pedometers and resistance bands to supplement the intervention.

TEMPO was designed based on three main frameworks, including the stress and coping framework [26], Bodenmann’s framework of dyadic coping [27], and self-efficacy theory [28]. Based on these frameworks and the main goal of TEMPO, a number of behavior change techniques (BCTs) were selected and implemented to help individuals adopt new behaviors and maintain them. A BCT is a distinct strategy that helps change or adjust the processes that govern behavior [29]. BCTs are important to consider when developing interventions because when BCTs are well delivered, they have greater potential to successfully change behavior [30] compared to passively or poorly integrated BCTs. BCTs are behavior specific [29]; for example, goal setting is used to set a specific behavioral target such as achieving a set number of minutes of physical activity per week or obtaining outcomes that can be derived from engaging in a set number of minutes of physical activity per week. However, once an individual learns how to use a BCT they may apply it to any behavior they wish to change [30]. BCTs included in previous behavioral interventions for men with prostate cancer, such as goal setting, self-monitoring, social support, instruction on how to perform the behavior, and adding objects to the environment, were integrated into TEMPO [31]. A total of 37 BCTs are implemented within TEMPO in various modules, worksheets, or health library factsheets (TEMPO was coded for BCTs, following the BCT Taxonomy (v1) [29], by two independent coders. See Table 1 for an overview of the BCTs implemented within TEMPO). The modules with associated worksheets comprise the main content of the intervention (includes 23 BCTs), with health library factsheets as additional supportive information (includes 14 BCTs). If dyads only complete the TEMPO modules, they will encounter 23 BCTs. The modules are each centered around 1-5 BCTs with the primary ones including goal setting, action planning, problem solving, self-monitoring, reviewing goals, and social support. For these primary BCTs, the module includes information about the BCTs, why they work, as well as worksheets the dyads can complete to learn how to implement the BCT. For example, in Module 2 which focuses on goal setting, dyads select two goals they want to achieve together and complete a worksheet to declare their goals. Depending on the path participants take through TEMPO (i.e., the tailored content they encounter), or the factsheets consulted in the health library participants may also encounter up to 14 additional BCTs. Many of these BCTs are simply described for the dyads so they can try them if they think they will be useful. 

Although BCTs are often included in behavior change interventions, researchers seldom describe their interventions with sufficient detail to determine the BCTs used, how they are implemented, and to the extent participants learned and engaged with the BCTs [31]. Identifying BCTs that help individuals successfully modify behavior, and understanding how they are used by participants, such as the dyads that take part in TEMPO, may be beneficial for assessing the acceptability of such a novel intervention and future interventions. Therefore, the purpose of this study is to explore how the participants describe their experiences using BCTs while enrolled in TEMPO.

## 2. Materials and Methods

### 2.1. Design

This study is a secondary data analysis of verbatim transcripts drawn from the primary qualitative study assessing the acceptability of TEMPO as part of Phase I [32]. Phase II, a pilot randomized controlled trial of TEMPO is underway (registered with Clinical Trials, ID: NCT04304196).

### 2.2. Recruitment Procedures

For the primary study, dyads were recruited through convenience sampling from four major hospitals or cancer centers across Canada. Clinicians identified patients that were potentially eligible to participate in the study and were referred to a research assistant to screen for eligibility. To be eligible to participate in the primary study, male participants at the time of recruitment must: (a) have had a confirmed prostate cancer diagnosis (localized or advanced) within the past two years, (b) have been undergoing or completed active treatment (i.e., surgery, chemotherapy, radiotherapy, and/or hormonotherapy; excludes watchful waiting), (c) have identified a primary caregiver willing to participate in the study, (d) have had access to the internet, and (e) read and understand English. Eligible caregivers were those identified by the patient as his primary source of support. Caregivers who were diagnosed with cancer in the previous year, receiving treatment for cancer, or have undergone any structured self-management program previously were excluded. Patients and caregivers also needed to be free from medical contraindications to participate in moderate physical activity. Dyads that were eligible to participate were provided with more details about the study by the research assistant who also assessed their interest in participating in the study. Interested dyads were emailed instructions about enrolling in TEMPO, whereby they could provide consent through TEMPO’s online platform. All participants from the primary study were eligible for this secondary analysis.

### 2.3. Data Collection

In the primary study, once consent was obtained and dyads were enrolled in TEMPO, they were encouraged to work through the five modules in TEMPO over the course of 10 weeks. Semi-structured interviews were conducted with dyads after completing Modules 1, 3, and 5. The interviews were conducted by different research assistants over the phone or in-person depending on the hospital where participants were recruited. All interviewers followed the same interview guides across hospital sites, with a different interview guide for each of the three interviews. In some instances, dyads were not available to complete all three interviews; therefore, interviews were combined to ensure all relevant and essential questions were asked from all three interview guides. The interview guides were designed to understand the dyads thoughts on usability, efficacy, content, and overall experience using TEMPO to assess the acceptability and usability of the intervention. The secondary analysis presented in this paper was to explore how dyads were discussing BCTs throughout TEMPO. Given the interview guides were not designed to focus on BCTs, participants were not probed about specific BCTs. For example, questions included: How has your experience been working towards your goal? What information was the most helpful so far? To what extent has this program helped you address the challenges you are facing? As dyads responded to these questions, they naturally discussed BCTs which provided a rich data set to be analyzed to understand the dyads’ experiences with BCTs. All interviews were audio recorded, transcribed verbatim, and managed in the NVivo software package, version 12 (QSR International Pty Ltd.) for analysis. Each participant was assigned an ID code that identified which hospital they were recruited from and whether they were the patient or caregiver. Any identifying data (i.e., names, locations) were removed to help ensure anonymity. Quotations from the participants were accompanied by their ID number, the interview number, and indicated whether it was the patient (PT) or caregiver (CG). 

### 2.4. Data Analysis

#### 2.4.1. Researcher Reflexivity

This secondary data analysis was conducted by two graduate students in Kinesiology (LH) and Nursing (KC) and the two principal investigators and creators of the TEMPO program (SL and LD) who are mid-career researchers in the fields of Nursing and Kinesiology, respectively. SL and LD have considerable experience conducting psychosocial and self-management research with cancer survivors. The two authors responsible for conducting the secondary thematic analysis (LH and KC) as well as the critical friend (LD) all completed an online BCT training [33], which contributes to the consistency and reliability of BCT coding and interpretation. 

#### 2.4.2. Analysis Approach

All data collected in the primary study (i.e., interviews from 19 dyads, spread across 46 separate transcripts) were included in this secondary analysis. The transcripts were analyzed first to identify BCTs using Michie’s BCT Taxonomy (v1) [29] then by an inductive thematic analysis to explore common experiences while using BCTs. The thematic analysis followed the six steps outlined by Braun and Clarke [34], which included familiarization with the data, generating initial codes, searching and reviewing themes, naming themes, and producing the final report. Two authors were involved with the coding process to identify common patterns and themes in the dyads’ experiences using BCTs throughout TEMPO. Four additional steps were taken to ensure quality standards, confirmability, credibility, and dependability. First, the senior author served as a critical friend throughout, and particularly during data analysis to help challenge and question coding that lead to further discussions. Secondly, the research assistants recruiting and communicating with the participants throughout TEMPO also conducted the multiple interviews, allowing for prolonged engagement with the dyads. Third, to ensure dependability, detailed descriptions of the steps taken during the research process and analysis were reported. Fourth, direct quotations were used from the dyads to depict their experiences and honor their voices and words. 

## 3. Results

### 3.1. Participant Demographics

A total of 19 patients with prostate cancer and their female caregiver were recruited, as a dyad, for the acceptability phase of TEMPO. The average age of patients was 64.7 years (SD = 7.22), and 62.3 years (SD = 6.62) for the caregivers. The dyads were most often married (*n* = 17) and living together (*n* = 18), where one patient identified as single. The majority of patients were diagnosed with early stage prostate cancer (*n* = 11), where surgery was the most common treatment (*n* = 12). More detailed socio-demographic information for patients and caregivers is presented in Table 2. 

### 3.2. Overview of Themes 

The dyads came across a number of BCTs while enrolled in TEMPO, and mainly discussed the BCTs that were the focus of an entire module. When a BCT was the main focus of a module, such as goal setting (Module 2) and social support (Module 4), dyads were exposed to and spent considerable time working with those BCTs. Additionally, dyads spoke about BCTs that appeared a number of times across different modules, such as reviewing goals. As the dyads discussed various BCTs, they described various contexts and relationships with BCTs, which is reflected in four common themes: (1) Learning new BCTs, (2) Engaging with BCTs learned in the past, (3) Resisting full engagement with BCTs, and (4) Experiencing positive outcomes from using BCTs. Theme 1 speaks to how the dyads learned about the BCTs, in detail, and how to properly apply them to their life and to various behaviors. The dyads discussed learning about effective goal setting and social support. Theme 2 involves dyads discussing how they had encountered various BCTs in the past, often in different contexts, such as in a work setting, and they applied those BCTs within the context of TEMPO. Theme 3 discusses how dyads did not always feel that applying the BCTs properly, or at all, was important for them, and showed some resistance to the idea of various BCTs. Theme 4 includes dyads expressing the positive outcomes that came from changing their behavior with the help of BCTs, such as achieving goals and feeling more confident. 

### 3.3. Theme 1: Learning New BCTs

#### 3.3.1. Goal Setting

Most dyads described their experiences learning BCTs for the first time and how to use them. For example, dyads were encouraged to set two goals at the start of TEMPO following the SMARTT goal principles, meaning goals should be specific, measurable, attainable, reasonable, timely, and together. One dyad expressed: 

That is helpful and the very idea of it, and also the idea of–that you have to be really specific. You can’t say I’m going to exercise more. No, you have to say what exercise you’re going to do, how often you’re going to do it, how much of it you’re going to do and when you’re going to do it and with whom and with what equipment. In other words, it says, setting a goal is not something nebulous. It has to be very specific, and emphasis on the specific. (PT, 11023-12023-2)

Another dyad said, “If I was setting my own goals I’d probably be not setting SMARTT goals, I’d be setting unattainable goals” (PT, 31051-32051-2). The dyads explained the importance of properly learning goal setting to effectively apply the technique and successfully achieve their goals. Another couple spoke about liking the experience of working through the goal setting worksheet, speaking to the importance of taking your time when creating goals: 

The SMARTT plan I thought was really good, how it explained everything and what it stood for. And I think the worksheets were good, like, it made us stop and think about, okay, how do you really–do you have anxiety, or stress, or this type … so yeah, I think they were all good to work through.” (CG, 31053-32053-2). 

TEMPO further explained the importance of reviewing goals over the course of the program. One patient described:

Well it was good that they–in the process it tells you not to give up and to reset your goals if you’ve set them too high, you know. So it’s encouraging…it’s a challenge, but like it says in the modules, you know, if you have to like look at your goals and reset them a little bit lower then do that. (PT, 31051-32051-2).

#### 3.3.2. Social Support

Module 4 of TEMPO is dedicated entirely to teaching dyads about the various sources of social support they can access and how they can use those sources of social support across all aspects of their lives. Within the module, five types of social support are described and examples of how each type of support can be used are provided. The instructions on BCTs related to social support helped the dyads understand how to effectively provide social support. One caregiver explained:

So, we took a week off. Well then after a week, it was a little–I said to him, maybe it’s time to go back, but I knew not to nag, you know, from reading some of the stuff in the module. It was an encouragement instead of a nag. (CG, 81018-82018-2)

The program further reminded dyads about the benefits and value of social support, and encouraged them to provide or seek more support. For one patient, learning the BCTs related to social support encouraged him to reach out for support to keep him active: 

I wasn’t keeping in contact with friends and so I reached out to them and now we’re-I’ve got some friends that I walk with-you know one person every Sunday, kind of thing. So I think the module triggered me to look for support but that’s-it wasn’t an area that I was really good at, to be honest with you. (PT, 81019-82019-3)

For another dyad, reading about social support reminded him this is a journey they are both going through, and to work together and support one another. 

So I think the thing about TEMPO that really struck me was that it’s not all about me, it’s the pair of us. It’s a challenge that both of us have to overcome. And quite often the spouse is the one that’s ignored in this. So it was a means for [partner’s name] and I to do something together. (PT, 31027-32017-1)

### 3.4. Theme 2: Engaging with BCTs Learned in the Past

Some dyads were aware of certain BCTs that they learned in different settings, such as at work or even through the media. For example, one caregiver said:

In my previous work there was a lot of goal-setting activities, I actually taught various types of goal setting as part of my work previously. So setting goals, understanding what your challenges are, and finding strategies to overcome those challenges is something that I’ve done instinctively for the time–almost 30 years–that I’ve been working. (PT, 31027-32027-1)

However, coming across the BCTs in the context of TEMPO taught the dyads how they can apply these techniques to aspects of their lives where they previously had not thought of applying them. “It’s a good reminder, the SMARTT goals, I’ve known about those for years and years, but I think we’re using them more now. You know, how much we’d like to do by when basically, I think that has helped. (CG, 31051-32051-2). Another dyad spoke about transferring the skills from one area of life to another: 

We just started working on Module 2 and I got the impression that it was going to be developing action plans for the goals in one. Now my initial reaction with the, you know, we both have worked as project managers so probably it will be a little bit repetitive or redundant. But you never know, sometimes when you get into something personal like this you don’t bring your skills that you use in other areas. I mean she’s one of the best project managers I’ve ever met but that doesn’t necessarily mean we can manage our own lives. (PT, 11007-12007-1)

TEMPO also supports the dyads in the actual application of the BCTs to encourage behavior change. One couple said: 

I found that–I mean I think the real value for [patient’s name] and I was the actual doing it, you know. So, you know, we know about SMARTT goals and action plans and stuff but we weren’t actually doing that. So, the value of the module was to actually make some goals and figure out how we were going to do them for Module 2. (CG, 11007-12007-2)

Overall, the dyads benefitted from learning, in detail, about BCTs and how they can apply them to help overcome challenges they face together as a result of the cancer diagnosis and experience. 

### 3.5. Theme 3: Resisting Full Engagement with BCTs 

There were dyads who, at times, did not feel the need to engage with the BCTs in the way in which it was presented, or did not see the benefit in the BCT altogether. For example, some dyads did not feel the need to write and complete the worksheets for goal setting or action planning. One patient expressed, “We didn’t write them. We looked at them and we did them orally together. We looked at the questions, you know, it was print them out and do them, and all that stuff, and we just thought, we don’t need to do that” (PT, 81018-82018-2). Another patient explained, “I’d set the goals in my head and so [the worksheet] didn’t make any difference, to be honest” (PT, 31027-32027-2). There were also dyads that did not feel certain BCTs would be relevant for them towards changing their behavior. For example, one dyad discussed difficulty working on their goal due to a lack of motivation and being busy with other projects. When prompted if they completed the problem-solving worksheets to help them overcome the challenges towards achieving their goal, the caregiver said “It wasn’t necessary” (CG, 11003-12003-2). One dyad failed to see the benefit of social support and completing goals together, where the caregiver expressed, “So far I have not found it useful. As I say, I was a bit surprised that, ‘Oh, they expect us to actually do exercises together?’ What a novel notion. I don’t know that we could manage any of these things” (CG, 11023-12023-1). Despite TEMPO describing how to successfully implement BCTs and their importance for changing behavior, some dyads were skeptical about their benefit.

### 3.6. Theme 4: Positive Outcomes as a Result of Using BCTs

As the dyads learned and successfully applied BCTs, they often spoke about changes in their behavior and the positive outcomes they experienced as a result. 

#### 3.6.1. Achieving Goals

As a result of creating goals, using action and coping planning, and adjusting goals when necessary, dyads expressed changes to their behavior. One patient expressed, “We’ve definitely done, you know a couple or three rounds in-in the basement with the exercise bands that we wouldn’t have done before” (PT, 31003-32003-2). Another dyad spoke about the benefit of exercising, which was a behavior change that resulted from following the BCTs within TEMPO: “So you get up in the morning and you go for a walk and you turn around and you do your stretching exercises, it does put you in a much better frame of mind.” (PT, 81018-82018-2) 

#### 3.6.2. Identifying and Managing Challenges

For some dyads, using BCTs, such as self-monitoring of behavior, allowed the dyads to incur a variety of benefits. In one instance, through self-monitoring, a caregiver was able to identify anxiety issues and seek help. 

One thing TEMPO helps me with, you know when we filled out the getting started, you know the graph, the one where it tells your moods and everything, I was extremely, extremely anxious…It did kind of push me to go the doctor–she put me on some antidepressants, a low dose…They have been almost a life changer. I wish I did this 40 years ago, but I was so averse to pills. It’s really made a big change in my life. (CG, 21037-22037-2). 

Another patient was having issues with fatigue, and his wife spoke about the benefits of using the BCT self-monitoring to assess fatigue levels: 

We wanted real details about where his fatigue was at so we picked logging his fatigue so that we could put some actuals against how he felt instead of, you know, when you’re having a bad day you say “Geez, I’ve felt bad for weeks,” you can actually go back and look at the fatigue log and see that you really were not feeling horrible for the last two weeks. So he’s been logging his fatigue levels so that he has more of an actual feeling for where he’s at, instead of how he felt at that moment. (CG, 81019-82019-2).

#### 3.6.3. Normalizing Challenges

One patient used social comparison from information provided in TEMPO to help normalize his feelings about depression. 

You read about it (signs of depression) and start to say well yeah some of these signs are mine, I mean that’s what was helpful about your program…Identifying that this is not an unusual thing, that this is a normal part of a healing process, normal part to feel this way after you’ve been through something major like cancer. (PT, 81018-82018-3).

#### 3.6.4. Feeling More Confident

Several dyads noted an increased confidence to achieve their goals after completing the program. One dyad explained:

We ended up with two goals achieved that we didn’t put down as goals and one was increase in self-esteem and we’ve had a long winter here, I don’t know if you know it, but we just woke up to snow again this morning. And let me tell you it just brings your spirits down […] but you go into your exercise room and you just do your exercises and you feel, “Okay I’ve accomplished one thing today, I checked that off, I’ve done one thing today.” So your self-esteem is certainly much better. (CG, 81018-82018-3) 

Another patient noted improved self-efficacy with problem solving and changing his mindset when faced with a barrier. 

I think I’ve used that a few times where I’ve fell off the wagon and wasn’t, for some reason, it rained and I injured myself and then it rained the next day and so I go, “oh my God, I haven’t walked in…”–I just reset-just reset the clock and felt good about it. Versus before I’d be beating myself up, like “oh my God, like you’ve fallen off the program here,” versus “this is okay, this has happened, for whatever reason, reset the clock and move forward again.” And so I felt comfortable doing that. (PT, 81019-82019-3)

## 4. Discussion

The purpose of this study was to explore prostate cancer survivor–caregiver dyads’ experiences using BCTs while enrolled in TEMPO. The dyads described learning how to effectively use certain BCTs, (re)engaging with BCTs they had learned in the past, and resisting full engagement with BCTs. As a result of using BCTs and adopting new behaviors, the dyads also spoke about the positive outcomes that resulted from behavior changes. Overall, the dyads were able to successfully implement the BCTs incorporated in TEMPO into their lives, and reported improvements in their quality of life.

The dyads primarily discussed engaging or reengaging with the BCTs that were the main focus of the modules, such as self-monitoring (Module 1), goal setting and action planning (Module 2), reviewing goals and problem solving (Module 3), and social support (Module 4). BCTs that were included as passive information in the health library were rarely or never discussed by the dyads in this study. It is apparent from our findings that when BCTs were taught/incorporated more thoroughly in TEMPO, the dyads were more likely to adopt them and subsequently achieve the benefits of the behavior change. This finding is consistent with previous literature, and presents an important practical implication of this study—it is important to effectively teach BCTs in order for them to be effective, as the passive implementation of BCTs may not be impactful [31,35,36,37]. 

TEMPO included a total of 37 BCTs. Participants who completed all the modules were certain to encounter 23 BCTs, and may encounter 14 additional BCTs depending on the path they chose through TEMPO and the health library information they access. Despite the many BCTs included in TEMPO, the participants only discussed six of them: self-monitoring, goal setting, action planning, reviewing goals, problem solving, and social support. Some researchers have explored the optimal number of BCTs to be included in an intervention and whether there is a relationship between the number of BCTs and the overall efficacy of the intervention. For example, following procedures developed by Gardiner et al. [35], Hallward et al. [31] coded the number of BCTs described in interventions that were deemed ‘promising’ or ‘non-promising’ (based on within- and between-subjects statistical comparisons on the target outcome variable) and compared the number of BCTs included in promising versus non-promising interventions. Hallward et al. [31] noted no significant differences in the number of BCTs included in promising versus non-promising interventions. Indeed, Michie et al. [37] and others [31,35,36] have demonstrated that the quantity of BCTs is less likely to impact the overall effectiveness of the intervention than the quality with which the BCTs are delivered. Therefore, interventions targeting behavior change should ensure that BCTs are delivered with high quality to maximize their potential impact. It should be noted that reviews exploring whether the number of BCTs included in an intervention is a relevant consideration have primarily been conducted on studies with one specific behavioral focus (e.g., increasing physical activity). TEMPO is distinct from these types of interventions because the dyads could have any number of behavioral foci, ranging from increasing physical activity, to implementing relaxation techniques, to better communication amongst the dyad. Given that there appears to be no risk of having additional BCT-related content in an intervention, it may be prudent to include more BCTs in interventions with broad behavioral foci. 

The participants enrolled in TEMPO came across as a relatively motivated group of individuals in the interviews, and perhaps already held strong intentions to work toward their behavior change goals. However, some dyads felt the BCTs were unnecessary, not relevant, or too much for them to manage. Researchers have acknowledged that some BCTs, such as action planning, are more than just techniques that are used to facilitate behavior change and are actually behaviors in themselves [29,38]. Thus, adopting BCTs to change a target behavior may, in fact, represent a relatively complex pattern of multiple behavior changes. Although multiple behavior change interventions have the potential to be effective [39], the complexity of making multiple behavior changes may also represent a considerable challenge to dyads who are already facing the burdens associated with cancer. Further research is needed to explore the perceived benefits and burdens of adopting BCTs in behavioral interventions. Other cases of resistance to engaging with BCTs were reflected by dyads who felt the activities included in the modules around certain BCTs were superfluous. These dyads opted to discuss the BCTs but not to complete the worksheets. The worksheets were provided in TEMPO to facilitate the learning of BCTs because previous research has indicated that engaging with BCTs more deliberately may yield more optimal behavioral outcomes [37]. When BCTs are learned and implemented in a dyadic fashion, however, discussing the BCT may represent sufficient engagement with the BCT for optimal outcomes to be achieved. Indeed, our findings indicate positive behavioral changes from dyads who took this approach. More research is needed to explore how BCTs can best be learned and engaged with to facilitate behavior change. 

This research should be interpreted within the context of its limitations. The goal to explore dyads’ experiences with BCTs is a secondary analysis of data from a qualitative study assessing the acceptability of TEMPO; thus, the interviews were not designed to specifically ask questions about BCTs. Data on the dyads’ interactions with BCTs throughout TEMPO were gleaned from unprompted discussions about BCTs. Although this limits a more rigorous investigation of BCTs, it provides an ecologically valid understanding of how the dyads learned and adopted these techniques. 

TEMPO was developed for dyads who require assistance dealing with the challenges of cancer survivorship. The participants enrolled in this study were a convenience sample of dyads who were dealing with subacute to chronic phases of prostate cancer and appeared to already be a relatively motivated group of individuals with interest in this type of program. In addition, the participants had relatively high levels of education, socioeconomic status, and participation in physical activity, and were often already actively working on addressing their challenges. Therefore, the findings from this research may not be generalizable to dyads facing worse social and cancer-related circumstances. Further research is needed to explore the experiences engaging with TEMPO among dyads with greater need for behavioral support. 

## 5. Conclusions

TEMPO was designed as one of the first web-based, self-management, psychosocial and physical activity programs to address the needs and challenges of prostate cancer patient–caregiver dyads. Dyads discussed their experiences working through TEMPO, using BCTs to help change their behaviors, overcome challenges, and lead to improved health outcomes. The dyads discussed learning BCTs, engaging with BCTs they had learned in other settings but had not applied to their challenges coping with cancer, and as a result, experienced positive outcomes from behavior changes. Some dyads resisted fully engaging with the BCTs, and TEMPO, but overall, all dyads found the program to be helpful for both the patient and the caregiver for addressing challenges and achieving better health outcomes. These findings highlight the quality in which BCTs were taught and incorporated within TEMPO, and how dyads can successfully use web-based programs to teach themselves how to use BCTs and change behavior in a self-directed manner.

## Figures and Tables

**Table 1:** Behavior Change Techniques incorporated into the TEMPO intervention.

Behavior Change Technique		Target Behavior	Example	Location in TEMPO *	Guaranteed to come across BCT while Using TEMPO? **
1.1	Goal setting (behavior)	Varies	Setting SMARTT goal based on dyads’ needs (goal can be for behavior or outcome)	Module 2	Yes
1.2	Problem solving	Varies	Instructed to create coping plans for set goals	Module 3	Yes
1.3	Goal setting (outcome)	Varies	Setting SMARTT goal based on dyads’ needs (goal can be for behavior or outcome)	Module 2	Yes
1.4	Action planning	Varies	Creating an action plan to achieve goals	Module 2	Yes
1.5	Review behavior goal(s)	Varies	When creating coping plans, can review and reset goals (goal can be for behavior or outcome)	Module 3	Yes
1.7	Review outcome goal(s)	Varies	When creating coping plan, can review and reset goals (goal can be for behavior or outcome)	Module 3	Yes
1.8	Behavioral contract	Varies	Signing and creating the SMARTT goal worksheet. You indicate “who” will complete the goal and write name.	Module 2	Yes
2.3	Self-monitoring of behavior	Varies	Using a symptom diary to record pain, fatigue, etc. (depends if monitoring behavior or outcomes)	Health library	No
2.4	Self-monitoring of outcome(s) of behavior	Varies	Using a symptom diary to record pain, fatigue, etc. (depends if monitoring behavior or outcomes)	Health library	No
3.1	Social support (unspecified)	Varies	Dyads write down examples of support provided in the past week	Module 4	Yes
3.2	Social support (practical)	Varies	Module explains sources of support and how they can help	Module 4	Yes
3.3	Social support (emotional)	Varies	Module explains sources of support and how they can help	Module 4	Yes
4.1	Instruction on how to perform the behavior	PA, relaxation techniques	Instructions how to do exercises, how to do deep breathing, muscle relaxation, etc.	Module 1, Health library	Yes
4.2	Information about antecedents	Varies	Provide information on events, situations, emotions, cognitions, that might predict behavior	Health library	No
5.1	Information about health consequences	Varies	Discussion around consequences of engaging or not engaging in certain behaviors (e.g., taking medication)	TEMPO intro, Health library	Yes
5.4	Monitoring of emotional consequences	Varies	Record in a diary emotions or feelings that can occur after communication issues, or performing another behavior	Health library	No
6.1	Demonstration of behavior	PA	Pictures and videos of exercises	Health library	
6.2	Social comparison	Varies	Using testimonials to show how others successfully used TEMPO to aid with new participants engagement with it	Each module Health Library	Yes
6.3	Information about others’ approval	Varies	Information from various professionals provided to explain what they think of behaviors	Health library	No
7.1	Prompts/cues	Varies	Putting action plans on fridge as a cue to complete goal	Module 2	Yes
8.1	Behavioral practice/rehearsal	PA	Encouraged to engage in physical activity multiple times a week, in different places, for different uses/benefits	Health library	No
8.2	Behavior substitution	PA	Replace sedentary time with active bouts	Module 1, Health library	Yes
8.4	Habit reversal	PA	Replacing sedentary time with active bouts, like when watching TV to move during commercials	Health library	No
8.6	Generalization of target behavior	Emotions, PA	Can practice deep breathing or muscle relaxation in any setting, any time	Health library	No
8.7	Graded tasks	PA	Encouraged to move up to harder exercises when they feel too easy	Health library	No
9.1	Credible source	Varies	Information, suggestions, content, etc. all developed by professionals in the field	All of TEMPO	Yes
9.2	Pros and cons	Making decisions	Making decisions about health care decisions/treatment	Module 1	Yes (but for not all behaviors)
10.7	Self-incentive	Varies	Set incentives for achieving each step in an action plan	Module 2	Yes
10.9	Self-reward	Varies	TOP TIP to reward self when competing goals	Module 2	Yes
11.1	Pharmacological support	Managing pain	Suggestion that pain can be controlled with medication	Health library	No
11.2	Reduce negative emotions	Emotions	Provides suggestions, like mindfulness, to reduce negative emotions	Health library	No
12.5	Adding objects to the environment	PA	Sending resistance bands and pedometers via mail	-	Yes
12.6	Body changes	Managing emotions, pain, symptoms	Using physical activity or relaxation techniques (altering body function) to help manage emotions, symptoms, pain, etc.	Module 1	Yes
13.2	Framing/reframing	Varies	Changing self-talk and perspective on waiting for treatment	Health Library	No
15.1	Verbal persuasion about capability	Varies	Told that successfully achieving goals is possible	Module 3	Yes
15.3	Focus on past success	Varies	Reflect and recall past successes throughout TEMPO	Module 5	Yes
15.4	Self-talk	Emotions	Using positive self-talk to manage negative emotions	Health library	No

**Note.** The numbers in the left-hand column indicate the behavior change technique (BCT) number from the Behavior Change Techniques Taxonomy. PA = Physical Activity. * The BCT may appear in more than one location, but only one location is indicated for simplicity. ** Although the dyads are guaranteed to come across the BCT if they complete the modules, they are not guaranteed to use the BCT.

**Table 2:** Socio-demographic data for participant dyads (*n* = 19).

	Caregivers (n = 17) * N (%)	Patients (n = 19) N (%)
Age (years)		
40–49	1 (5.9%)	1 (5.3%)
50–59	4 (23.5%)	2 (10.5%)
60–69	9 (52.9%)	11 (57.9%)
≥70	3 (17.7%)	5 (26.3%)
Mean	62.3	64.7
SD	6.62	7.22
Median	63	66
Language		
English	15 (88.2%)	16 (18.2%)
French	1 (5.9%)	1 (5.3%)
Other	1 (5.9%)	2 (10.5%)
Education		
High School	2 (11.8%)	1 (5.3%)
Post-secondary diploma	4 (23.5%)	7 (36.8%)
Undergraduate degree	11 (64.7%)	6 (31.6%)
Master’s Degree	0 (0%)	3 (15.8%)
Doctorate Degree	0 (0%)	2 (10.5%)
Health Conditions		
Arthritis	6 (35.3%)	6 (31.6%)
Diabetes	2 (11.8%)	7 (36.8%)
Emotional problems	5 (29.4%)	5 (26.4%)
Hypertension	4 (23.5%)	9 (47.4%)
Heart Problems	1 (5.9%)	5 (26.4%)
Intestinal polyps	1 (5.9%)	3 (15.8%)
Liver disease	1 (5.9%)	2 (10.5%)
Sleep Apnea	1 (5.9%)	5 (26.4%)
Stomach problems	2 (11.8%)	3 (15.8%)
Thyroid	2 (11.8%)	3 (15.8%)
Household Income (n = 19 dyads)		
USD 40,000 to USD 59,999	3 (15.8%)	
USD 60,000 to USD 79,999	1 (5.3%)	
USD 80,000 to USD 99,999	4 (21.0%)	
≥ USD 100,000	7 (36.8%)	
Prefer not to answer	4 (21.0%)	
Marital Status (n = 19 dyads)		
Single	1 (5.3%)	
Married	17 (89.4%)	
Common Law	1 (5.3%)	
Patient Cancer Characteristics		
Years since prostate cancer diagnosis		
<1 year		4 (21.0%)
1–3 years		10 (52.6%)
3–5 years		5 (26.4%)
Stage of Cancer at diagnosis		
Early Stage		11 (57.9%)
Advanced Stage		6 (31.6%)
Unknown		2 (10.5%)
Cancer treatments receiving, received, or plan to receive		
Surgery		12 (63.2%)
Chemotherapy		4 (21.0%)
Radiotherapy		10 (52.6%)
Hormone Treatment		7 (36.8%)
Brachytherapy		2 (10.5%)
Watchful waiting		2 (10.5%)
Other		1 (5.3%)

* Caregiver data only available for 17/19 dyads. One patient was interviewed alone (e.g., his caregiver participated in TEMPO but not in the interviews), while the remaining CG data is missing.

## References

[1] Canadian Cancer Statistics Advisory Committee Canadian Cancer Statistics 2019. http://cancer.ca/Canadian-Cancer-Statistics-2019-EN.

[2] American Cancer Society (2020). Cancer Facts & Figures 2020.

[3] National Coalition for Cancer Survivorship Defining Cancer Survivorship. https://www.canceradvocacy.org/news/defining-cancer-survivorship/.

[4] Bourke L., Homer Kate E., Thaha Mohamed A., Steed L., Rosario Derek J., Robb Karen A., Saxton John M., Taylor Stephanie J.C. (2013). Interventions for promoting habitual exercise in people living with and beyond cancer. Cochrane Database Syst. Rev..

[5] Hsiao C.P., Loescher L.J., Moore I.M. (2007). Symptoms and symptom distress in localized prostate cancer. Cancer Nurs..

[6] Taylor L.G., Canfield S.E., Du X.L. (2009). Review of major adverse effects of androgen-deprivation therapy in men with prostate cancer. Cancer.

[7] Donovan K.A., Walker L.M., Wassersug R.J., Thompson L.M.A., Robinson J.W. (2015). Psychological effects of androgen-deprivation therapy on men with prostate cancer and their partners. Cancer.

[8] Casey R.G., Corcoran N.M., Larry Goldenberg S. (2012). Quality of life issues in men undergoing androgen deprivation therapy: A review. Asian J. Androl..

[9] Chipperfield K., Fletcher J., Millar J., Brooker J., Smith R., Frydenberg M., Burney S. (2013). Predictors of depression, anxiety and quality of life in patients with prostate cancer receiving androgen deprivation therapy. Psychooncology.

[10] Sinha M. (2013). Spotlight on Canadians: Results from the General Social Survey. Portrait of Caregivers 2012. Stat. Canada.

[11] DuBenske L.L., Wen K.-Y., Gustafson D.H., Guarnaccia C.A., Cleary J.F., Dinauer S.K., McTavish F.M. (2008). Caregivers’ differing needs across key experiences of the advanced cancer disease trajectory. Palliat. Support. Care.

[12] Kim Y., Schulz R. (2008). Family caregivers’ strains: Comparative analysis of cancer caregiving with dementia, diabetes, and frail elderly caregiving. J. Aging Health.

[13] Stenberg U., Ruland C., Miaskowski C. (2010). Review of the literature on the effects of caring for a patient with cancer. Psychooncology.

[14] Palos G.R., Mendoza T.R., Liao K.P., Anderson K.O., Garcia-Gonzalez A., Hahn K., Nazario A., Ramondetta L.M., Valero V., Lynch G.R. (2011). Caregiver symptom burden: The risk of caring for an underserved patient with advanced cancer. Cancer.

[15] Resendes L.A., McCorkle R. (2006). Spousal responses to prostate cancer: An integrative review. Cancer Investig..

[16] Badr H., Lipnick D., Diefenbach M.A., Posner M., Kotz T., Miles B., Genden E. (2016). Development and usability testing of a web-based self-management intervention for oral cancer survivors and their family caregivers. Eur. J. Cancer Care (Engl.).

[17] Northouse L.L., Katapodi M.C., Song L., Zhang L., Mood D.W. (2010). Interventions with family caregivers of cancer patients: Meta-analysis of randomized trials. CA Cancer Cournal Clin..

[18] Regan T.W., Lambert S.D., Girgis A., Kelly B., Kayser K., Turner J. (2012). Do couple-based interventions make a difference for couples affected by cancer? A systematic review. BMC Cancer.

[19] Thorsen L., Courneya K.S., Stevinson C., Fosså S.D. (2008). A systematic review of physical activity in prostate cancer survivors: Outcomes, prevalence, and determinants. Support. Care Cancer.

[20] Bourke L., Smith D., Steed L., Hooper R., Carter A., Catto J., Albertsen P.C., Tombal B., Payne H.A., Rosario D.J. (2016). Exercise for men with prostate cancer: A systematic review and meta-analysis. Eur. Urol..

[21] Keogh J.W.L., MacLeod R.D. (2012). Body composition, physical fitness, functional performance, quality of life, and fatigue benefits of exercise for prostate cancer patients: A systematic review. J. Pain Symptom Manag..

[22] Baumann F.T., Zopf E.M., Bloch W. (2012). Clinical exercise interventions in prostate cancer patients-a systematic review of randomized controlled trials. Support. Care Cancer.

[23] Swartz J.J., Keir S.T. (2007). Program Preferences to Reduce Stress in Caregivers of Patients With Brain Tumors. Clin. J. Oncol. Nurs..

[24] Lambert S.D., Duncan L.R., Kapellas S., Bruson A.M., Myrand M., Santa Mina D., Culos-Reed N., Lambrou A. (2016). A descriptive systematic review of physical activity interventions for caregivers: Effects on caregivers’ and care recipients’ psychosocial outcomes, physical activity levels, and physical health. Ann. Behav. Med..

[25] Martin A.C., Keats M.R. (2014). The impact of yoga on quality of life and psychological distress in caregivers for patients with cancer. Oncol. Nurs. Forum.

[26] Lazarus R.S., Folkman S. (1984). Stress, Appraisal, and Coping.

[27] Bodenmann G. (1995). A systemic-transactional conceptualization of stress and coping in couples. Swiss J. Psychol. Zeitschrift für Psychol..

[28] Bandura A. (1986). Social Foundations of Thought and Action: A Social Cognitive Theory.

[29] Michie S., Richardson M., Johnston M., Abraham C., Francis J., Hardeman W., Eccles M.P., Cane J., Wood C.E. (2013). The behavior change technique taxonomy (v1) of 93 hierarchically clustered techniques: Building an international consensus for the reporting of behavior change interventions. Ann. Behav. Med..

[30] Michie S., West R., Sheals K., CA G. (2018). Evaluating the effectiveness of behavior change techniques in health-related behavior: A scoping review of methods used. Transl. Behav. Med..

[31] Hallward L., Patel N., Duncan L.R. (2018). Behavior change techniques in physical activity interventions for men with prostate cancer: A systematic review. J. Health Psychol..

[32] Lambert S.D., Duncan L.R., Ellis J., Schaffler J., Loban E., Robinson J.W., Culos-Reed S.N., Matthew A., Clayberg K., Santa Mina D. Acceptability and usefulness of a dyadic, tailored, web-based, psychosocial and physical activity self-management programme (TEMPO): A qualitative study. J. Clin. Med..

[33] Wood C.E., Richardson M., Johnston M., Abraham C., Francis J., Hardeman W., Michie S. (2015). Applying the behaviour change technique (BCT) taxonomy v1: A study of coder training. Transl. Behav. Med..

[34] Braun V., Clarke V. (2006). Using thematic analysis in psychology. Qual. Res. Psychol..

[35] Gardner B., Smith L., Lorencatto F., Hamer M., Biddle S.J.H. (2016). How to reduce sitting time? A review of behavior change strategies used in sedentary behavior reduction interventions among adults. Health Psychol. Rev..

[36] Martin J., Chater A., Lorencatto F. (2013). Effective behavior change techniques in the prevention and management of childhood obesity. Int. J. Obes..

[37] Michie S., Abraham C., Whittington C., McAteer J., Gupta S. (2009). Effective techniques in healthy eating and physical activity interventions: A meta-regression. Heal. Psychol..

[38] Sweet S.N., Brawley L.R., Hatchell A., Gainforth H.L., Latimer-Cheung A.E. (2014). Can Persuasive Messages Encourage Individuals to Create Action Plans for Physical Activity?. J. Sport Exerc. Psychol..

[39] Prochaska J., Prochaska J. (2011). A Review of Multiple Health Behavior Change Interventions for Primary Prevention. Am. J. Lifestyle Med..

